# Magnetic neutron scattering from spherical nanoparticles with Néel surface anisotropy: atomistic simulations

**DOI:** 10.1107/S1600576722008949

**Published:** 2022-11-04

**Authors:** Michael P. Adams, Andreas Michels, Hamid Kachkachi

**Affiliations:** a Department of Physics and Materials Science, University of Luxembourg, 162A avenue de la Faiencerie, L-1511 Luxembourg, Grand Duchy of Luxembourg; b Laboratoire PROMES CNRS UPR8521, Université de Perpignan via Domitia, Rambla de la Thermodynamique, Tecnosud, F-66100 Perpignan, France; Australian Nuclear Science and Technology Organisation, Lucas Heights, Australia

**Keywords:** magnetic neutron scattering, small-angle neutron scattering, magnetic nanoparticles, surface anisotropy, micromagnetics

## Abstract

A dilute ensemble of randomly oriented non-interacting spherical nanomagnets is considered, and its magnetization structure and ensuing neutron scattering response are investigated by numerically solving the Landau–Lifshitz equation. Taking into account the isotropic exchange interaction, an external magnetic field, a uniaxial magnetic anisotropy for the particle core, and in particular the Néel surface anisotropy, the magnetic small-angle neutron scattering cross section and pair-distance distribution function are calculated from the obtained equilibrium spin structures. The numerical results are compared with the well known analytical expressions for uniformly magnetized particles and provide guidance to the experimentalist. In addition, the effect of a particle-size distribution function is modelled.

## Abstract 2

Based on the Landau–Lifshitz equation, atomistic simulations of the magnetic neutron scattering from inhomogeneously magnetized spherical nanoparticles with a strong surface anisotropy are carried out.

## 1. Introduction

Magnetic nanoparticles are the subject of intense worldwide research efforts which are partly motivated by potential applications in areas such as medicine, biology and nanotechnology [see *e.g.* Lak *et al.* (2021 ▸), Diebold & Calonge (2010 ▸), De *et al.* (2008 ▸), Baetke *et al.* (2015 ▸), Stark *et al.* (2015 ▸), Han *et al.* (2019 ▸), Batlle *et al.* (2022 ▸) and references therein]. In the majority of studies, the internal spin structure of the nanoparticles is neglected and assumed to be uniform (called the macro- or superspin model). While this is probably justified in many application-oriented approaches in which an overall understanding is sufficient, it is of interest, at least from the standpoint of fundamental science, to elucidate the effect of a non-uniform spin structure on a certain physical property.

Scattering techniques, in particular employing X-rays and neutrons, have proved to be very powerful in this endeavour, since they provide statistically averaged information on a large number of scattering particles. For instance, using Monte Carlo simulations of a discrete atomistic spin model, Köhler *et al.* (2021 ▸) have numerically studied the influence of antiphase boundaries in iron oxide nanoparticles on their spin structure. These authors used the Debye scattering equation to relate the internal spin disorder to the broadening of certain X-ray Bragg peaks. Vivas *et al.* (2020 ▸) carried out micromagnetic continuum calculations of the spin structure of defect-free iron nanoparticles and related a vortex-type magnetization configuration to certain signatures in the magnetic neutron scattering cross section and correlation function.

Magnetic small-angle neutron scattering (SANS) is a powerful technique for investigating spin structures on the mesoscopic length scale (∼1–100 nm) and inside the volume of magnetic materials (Mühlbauer *et al.*, 2019 ▸; Michels, 2021 ▸). Recent SANS studies of magnetic nanoparticles, in particular employing spin-polarized neutrons, unanimously demonstrate that their spin textures are highly complex and exhibit a variety of non-uniform, canted or core–shell-type configurations [see *e.g.* Disch *et al.* (2012 ▸), Krycka *et al.* (2014 ▸), Hasz *et al.* (2014 ▸), Günther *et al.* (2014 ▸), Maurer *et al.* (2014 ▸), Dennis *et al.* (2015 ▸), Grutter *et al.* (2017 ▸), Oberdick *et al.* (2018 ▸), Ijiri *et al.* (2019 ▸), Bender *et al.* (2019 ▸), Bersweiler *et al.* (2019 ▸), Zákutná *et al.* (2020 ▸), Honecker *et al.* (2022 ▸) and references therein]. The analysis of magnetic SANS data relies largely on structural form-factor models for the cross section, borrowed from nuclear SANS, which do not properly account for the existing spin inhomogeneity inside a magnetic nanoparticle. Progress in magnetic SANS theory (Honecker & Michels, 2013 ▸; Michels *et al.*, 2014 ▸; Mettus & Michels, 2015 ▸; Erokhin *et al.*, 2015 ▸; Metlov & Michels, 2015 ▸, 2016 ▸; Michels *et al.*, 2016 ▸, 2019 ▸; Mistonov *et al.*, 2019 ▸; Zaporozhets *et al.*, 2022 ▸) strongly suggests that, for the analysis of experimental magnetic SANS data, the spatial nanometre-scale variation of the orientation and magnitude of the magnetization vector field must be taken into account, going beyond the macrospin-based models that assume a uniform magnetization.

In this paper, we employ atomistic simulations using the Landau–Lifshitz equation (LLE) to investigate the role of the Néel surface anisotropy in magnetic nanoparticles and its effect on the magnetic SANS cross section and correlation function. We take into account the isotropic exchange interaction, an external magnetic field, a magnetocrystalline anisotropy for the core of the nanoparticles and Néel anisotropy for spins on the surface. The influence of a particle-size distribution function on the magnetic SANS cross section and pair-distance distribution function is also studied. The numerical results reveal marked differences from the superspin model and provide guidance for the experimentalist to identify non-uniform spin structures inside magnetic nanoparticles. We also refer to our accompanying analytical study of the problem (Adams *et al.*, 2022 ▸), which is restricted to a linear approximation in the magnetization deviation.

The paper is organized as follows. In Section 2 we provide information on the atomistic simulations using the LLE. In Section 3 we display the expressions for the magnetic SANS cross section and for the pair-distance distribution function. The results of the numerical calculations are discussed in Section 4, with Section 4.1 focusing on the effect of the Néel surface anisotropy and Section 4.2 discussing the influence of a log-normal particle-size distribution on the SANS observables. Section 5 summarizes the main findings of this study and provides an outlook on future challenges. Appendix *A* [App appa] features results for the SANS observables for different sign combinations of the anisotropy constants.

## 2. Details of the atomistic SANS modelling using the Landau–Lifshitz equation

**Figure 1:**
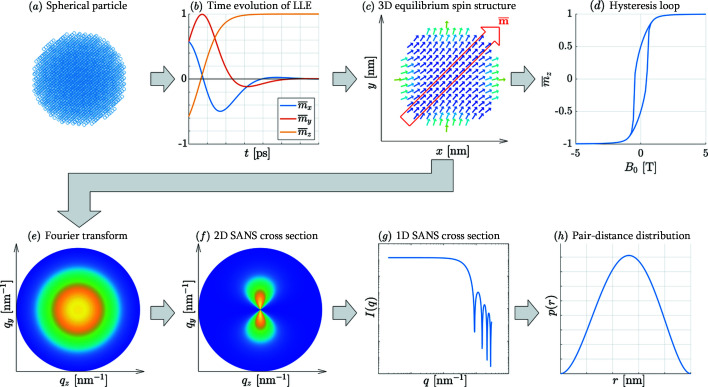
A flow chart explaining the atomistic SANS simulation procedure. (*a*) A spherical nanoparticle is cut from a simple cubic grid with *N* × *N* × *N* atoms. (*b*) The time evolution of the Cartesian magnetization components obtained by solving the Landau–Lifshitz equation. (*c*) The computed equilibrium spin structure of a spherical nanoparticle at remanence (cut through the centre of the particle). (*d*) A hysteresis loop of an ensemble of randomly oriented nanoparticles. (*e*) The computed Fourier transform. (*f*) The two-dimensional magnetic SANS cross section dΣ_M_/dΩ. (*g*) The azimuthally averaged magnetic SANS cross section *I*(*q*). (*h*) The pair-distance distribution function *p*(*r*).

Fig. 1 ▸ shows a schematic depiction of the procedure adopted here to generate and calculate the spin structure, and to obtain the ensuing magnetic SANS cross section and correlation function. This flow-chart-type representation will be discussed in more detail below.

A spherical many-spin nanomagnet is viewed as a crystallite consisting of 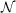 atomic magnetic moments **μ**_*i*_ = μ_a_ **m**_*i*_, where μ_a_ denotes the magnitude of the atomic magnetic moment and **m**_*i*_ is a unit vector specifying its orientation. We assume the spins ‘sit’ on a simple cubic lattice, so that μ_a_ = *M*_s_ *a* ^3^, where *M*_s_ is the saturation magnetization of the material and *a* is the lattice constant. The spherical shape of the nanomagnet is cut from a simple cubic regular grid [Fig. 1 ▸(*a*)] and its radius *R* is defined as 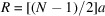 , where the integer *N* is the number of atoms on the side of the cubic grid. The magnetic state of the nanomagnet is investigated with the help of the atomistic approach based on the following Hamiltonian (Dimitrov & Wysin, 1994 ▸; Kodama & Berkovitz, 1999 ▸; Kachkachi & Garanin, 2001*a* ▸,*b* ▸; Iglesias & Labarta, 2001 ▸; Kachkachi & Dimian, 2002 ▸; Kachkachi & Garanin, 2005 ▸; Kazantseva *et al.*, 2008 ▸): 

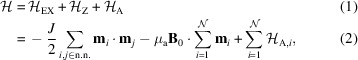

where 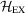 is the nearest-neighbour (n.n.) exchange energy, *J* > 0 is the exchange parameter, 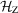 denotes the Zeeman energy, **B**_0_ is the homogeneous externally applied magnetic field and 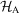 represents the magnetic anisotropy energy. For the core spins we assume the anisotropy to be of uniaxial symmetry, while for surface spins we adopt the model proposed by Néel (1954 ▸). 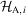 can then be expressed as 

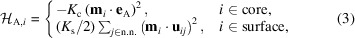

where *K*_c_ > 0 and *K*_s_ > 0 denote, respectively, the core and surface anisotropy constants, **e**_A_ is a unit vector along the core anisotropy easy direction, and **u**_*ij*_ = (**r**_*i*_ − **r**_*j*_)/∥**r**_*i*_ − **r**_*j*_∥ is a unit vector connecting the nearest-neighbour spins *i* and *j*. The surface spins are defined as those spins which have a coordination number less than six.

The magnetodipolar interaction has been ignored in our simulations. This is motivated by the numerical complexity of this energy term, in particular for atomistic simulations (here, for a 10 nm diameter particle the number of spins is 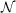 = 11 633), and by the expectation that it is of minor relevance for smaller-sized nanomagnets (Köhler *et al.*, 2021 ▸; Pathak & Hertel, 2021 ▸).

The dynamics of each individual magnetic moment **m**_*i*_ are described by the Landau–Lifshitz equation (LLE) (Berkov, 2007 ▸),

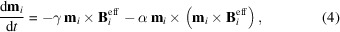

where γ is the gyromagnetic ratio and α denotes the damping constant. The deterministic effective magnetic field acting on the spin *i* is given by

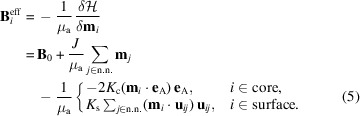

The LLE is solved numerically by using the explicit Euler forward-projection method (Baňas, 2005 ▸), which consists of two steps. The first step, as seen from equation (6) below, is the simple Euler forward scheme, and the second step, as seen from equation (7), is the projection (or normalization) onto the unit sphere to enforce the constraint ∥**m**_*i*_∥ = 1. Since we are interested in the static equilibrium, this first-order method is fully appropriate. In equations (6) and (7), *k* is the time iteration index while *i* refers to the *i*th lattice site, 

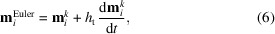




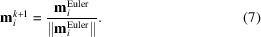


*h*_t_ denotes the time step for the integration procedure. For the termination of the energy minimization, we employ the following criterion: 

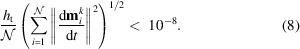

The macroscopic state of the nanomagnet is then described by the following super- or macrospin (representing the net magnetic moment): 

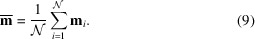

As an example, we show in Fig. 1 ▸(*b*) the temporal evolution of the Cartesian magnetization components of 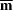 and in Fig. 1 ▸(*c*) the numerically computed equilibrium spin configuration for a spherical nanomagnet at zero applied field in a plane across its centre. It is seen that the spins at the centre of the nanoparticle are directed along 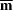 while the surface spins exhibit significant misalignment, which is due to the presence of the Néel surface anisotropy. Note that **m**_*i*_ are unit vectors, whereas generally 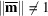 .

In our simulations we use the following parameters: atomic magnetic moment μ_a_ = 1.577 × 10^−23^ A m^2^ (corresponding to 1.7 μ_B_ with μ_B_ the Bohr magneton), lattice constant *a* = 0.3554 nm, *M*_s_ = μ_a_/*a* ^3^ = 351 kA m^−1^, exchange constant *J* = 8.7 × 10^−22^ J atom^−1^, core anisotropy constant *K*_c_ = 3 × 10^−24^ J atom^−1^, damping constant α = 3 × 10^11^ s^−1^ T^−1^, gyromagnetic constant γ = 1.76 × 10^11^ s^−1^ T^−1^ and an integration time step of *h*_t_ = 5 fs. The surface anisotropy constant *K*_s_ was used as an adjustable parameter. Experimental *K*_s_ values for nanoparticles and thin films can be found in the work of Gradmann (1986 ▸), Batlle *et al.* (2022 ▸) and O’Handley (2000 ▸). A value of *K*_s_ = 5.22 × 10^−21^ J atom^−1^ has been estimated by Kachkachi & Dimian (2002 ▸) for a 4 nm-sized face-centred cubic cobalt particle.

For the calculation of the magnetic SANS cross section dΣ_M_/dΩ [Fig. 1 ▸(*f*)], it is necessary to compute the discrete Fourier transform of all the **m**_*i*_ belonging to the spherical nanomagnet [Fig. 1 ▸(*e*)]. In Section 3, the expressions for dΣ_M_/dΩ are formulated for a continuous magnetization distribution **M**(**r**) and of its Fourier transform 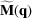 . These functions are defined as follows: 

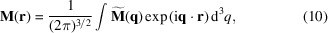




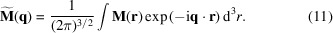

**Figure 2:**
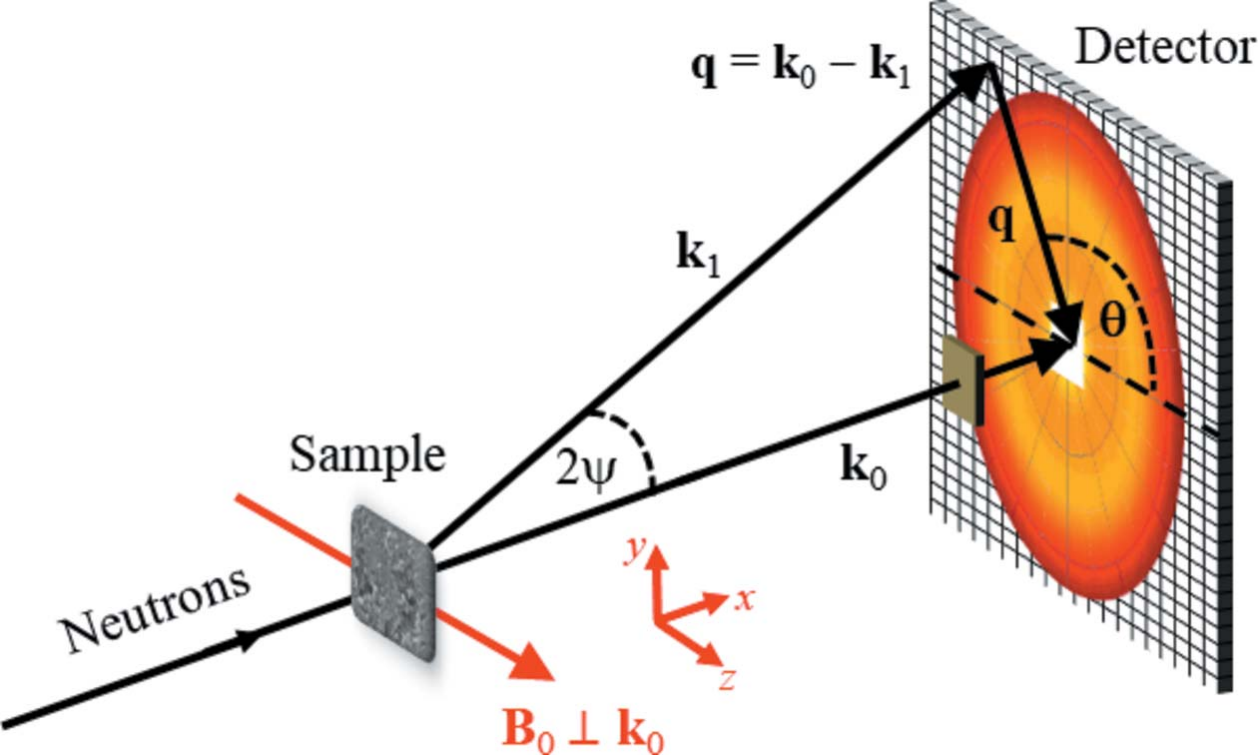
A sketch of the neutron scattering geometry. The applied magnetic field **B**_0_ ∥ **e**_*z*_ is perpendicular to the wavevector **k**_0_ ∥ **e**_*x*_ of the incident neutron beam (**B**_0_ ⊥ **k**_0_). The momentum transfer or scattering vector **q** is defined as the difference between **k**_0_ and **k**_1_, *i.e.* **q** = **k**_0_ − **k**_1_. SANS is usually implemented as elastic scattering (*k*_0_ = *k*_1_ = 2π/λ) and the component of **q** along the incident neutron beam, here *q*_*x*_, is much smaller than the other two components, so that 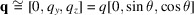 . This demonstrates that SANS predominantly probes correlations in the plane perpendicular to the incident beam. For elastic scattering, the magnitude of **q** is given by 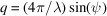 , where λ denotes the mean wavelength of the neutrons and 2ψ is the scattering angle. The angle θ = ∠(**q**, **B**_0_) is used to describe the angular anisotropy of the recorded scattering pattern on the two-dimensional position-sensitive detector.

Using **μ**_*i*_ = μ_a_ **m**_*i*_, the discrete-space Fourier transform is computed as

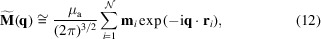

where **r**_*i*_ is the location point of the *i*th spin and **q** represents the wavevector (scattering vector, defined in Fig. 2 ▸). Equation (12) establishes the relation between the outcome of the simulations, **m**_*i*_, and the magnetic SANS cross section, dΣ_M_/dΩ. In the standard SANS geometry, the **q** space of interest is defined by **q** = *q*[0, sinθ, cosθ], which corresponds to the two-dimensional detector plane (*q*_*x*_ = 0, see Fig. 2 ▸). The two- and one-dimensional magnetic SANS cross sections dΣ_M_/dΩ [Figs. 1 ▸(*f*) and (*g*), respectively] are then computed according to equation (13). A further Fourier transformation yields the pair-distance distribution function [Fig. 1 ▸(*h*)].

**Figure 3:**
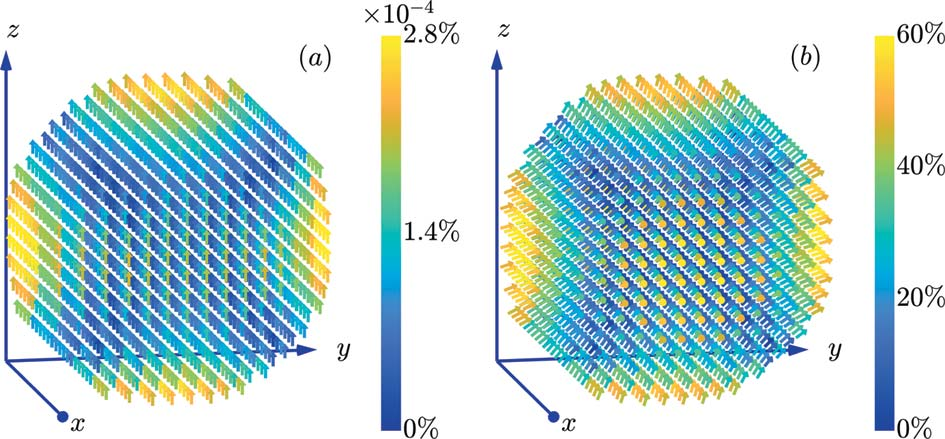
Selected 3D equilibrium spin structures arising from the Néel surface anisotropy [compare also with Figs. 2 and 3 in the accompanying analytical study (Adams *et al.*, 2022 ▸)]. (*a*) *K*_s_ = 5.22 × 10^−23^ J atom^−1^ and (*b*) *K*_s_ = 52.2 × 10^−23^ J atom^−1^. Further parameters are core-anisotropy axis **e**_A_ = [0, 0, 1], core-anisotropy constant *K*_c_ = 3 × 10^−24^ J atom^−1^ and external magnetic field **B**_0_ = [0, 0, 150 mT]. The particle diameter is *D* = 5 nm. The colour code depicts the spin misalignment relative to the average magnetization vector, namely δ*m*_*j*_ = 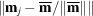 . At the surface of the nanomagnet the spin deviations are larger than those in the core.

At each value of the external field, atomistic simulations of the spin structure and of the ensuing magnetic SANS cross section were carried out for 256 random orientations of the core anisotropy axes **e**_A_ of the particle with respect to the field **B**_0_. More specifically, once the lattice orientation has been randomly selected, the easy-axis orientation of the particle’s core and the distribution of the Néel anisotropy are fixed. The whole system (core plus surface anisotropy) is then randomly rotated relative to **B**_0_. For the generation of the random angles, we used the low-discrepancy *Sobol* sequence (*sob*, https://www.mathworks.com/help/stats/sobolset.html). Therefore, except Fig. 3, all the data shown in this paper correspond to an ensemble of randomly oriented particles. The simulations were carried out by starting from a large positive (saturating) field of about 10 T, and then the field was reduced in steps of, typically, 30 mT.

## 3. Magnetic SANS cross section and pair-distance distribution function

The quantity of interest in experimental SANS studies is the elastic magnetic differential scattering cross section dΣ_M_/dΩ, which is usually recorded on a two-dimensional position-sensitive detector. For the most commonly used scattering geometry in magnetic SANS experiments, where the applied magnetic field **B**_0_ ∥ **e**_*z*_ is perpendicular to the wavevector **k**_0_ ∥ **e**_*x*_ of the incident neutrons (see Fig. 2 ▸), dΣ_M_/dΩ (for un­polarized neutrons) can be written as (Mühlbauer *et al.*, 2019 ▸)

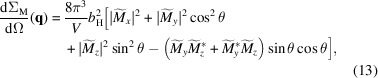

where *V* is the scattering volume, *b*_H_ = 2.91 × 10^8^ A^−1^ m^−1^ is the magnetic scattering length in the small-angle regime (the atomic magnetic form factor is approximated by 1 since we are dealing with forward scattering), 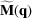 = 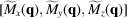 represents the Fourier transform of the magnetization vector field **M**(**r**) = [*M*_*x*_(**r**), *M*_*y*_(**r**), *M*_*z*_(**r**)], θ denotes the angle between **q** and **B**_0_, and the asterisk * stands for the complex-conjugated quantity. Note that in the perpendicular scattering geometry the Fourier components are evaluated in the plane *q*_*x*_ = 0 (see Fig. 2 ▸).

The numerically computed magnetic SANS cross sections that are displayed in this paper correspond to the following average: 

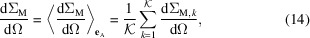

where dΣ_M, *k*_/dΩ represents (for fixed *K*_s_ and *B*_0_) the magnetic SANS cross section for a particular core easy-axis orientation **e**_A_ (with reference to the index ‘*k*’) and 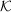 denotes the number of random configurations. Equation (14) implies the absence of interparticle interactions.

For a uniformly magnetized spherical particle with its saturation direction parallel to **e**_*z*_, *i.e.* *M*_*x*_ = *M*_*y*_ = 0 and *M*_*z*_ = *M*_s_, equation (13) reduces to

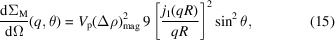

where *V*_p_ = 4π*R* ^3^/3 is the particle’s volume, 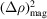 = 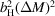 = 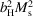 is the magnetic scattering-length density contrast and *j*_1_(*qR*) is the first-order spherical Bessel function. The well known analytical result for the homogeneous sphere case [equation (15)] and its correlation function [see equation (18) below] serve as a reference for comparison with the non-uniform case.

It is often convenient to average the two-dimensional SANS cross section (dΣ_M_/dΩ)(**q**) = (dΣ_M_/dΩ)(*q*_*y*_, *q*_*z*_) = (dΣ_M_/dΩ)(*q*, θ) along certain directions in **q** space, *e.g.* parallel (θ = 0) or perpendicular (θ = π/2) to the applied magnetic field, or even over the full angular θ range. In the following, we consider the 2π azimuthally averaged magnetic SANS cross section, 

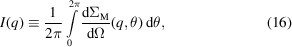

which is used to compute the pair-distance distribution function *p*(*r*) according to

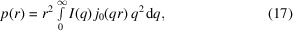

where 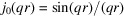 is the spherical Bessel function of zero order. *p*(*r*) corresponds to the distribution of real-space distances between volume elements inside the particle weighted by the excess scattering-length density distribution; see the reviews by Glatter (1982 ▸) and Svergun & Koch (2003 ▸) for detailed discussions of the properties of *p*(*r*) and for information on how to compute it by indirect Fourier transformation (Bender *et al.*, 2017 ▸). For our discrete simulation data, the integrals in equations (16) and (17) were approximated by the trapezoidal rule. Apart from constant prefactors, *p*(*r*) of the azimuthally averaged single-particle cross section [equation (15)], corresponding to a uniform sphere magnetization, is given by (for *r* ≤ 2*R*)

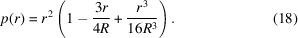

We also display results for the correlation function *c*(*r*), which is related to *p*(*r*) by 

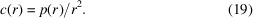

As we will demonstrate in the following, when the particles’ spin structure is inhomogeneous, dΣ_M_/dΩ and the corresponding *p*(*r*) and *c*(*r*) differ significantly from the homogeneous case [equations (15) and (18)], which serves as a reference. Because of the *r* ^2^ factor, features in *p*(*r*) at medium and large distances are more pronounced than those in *c*(*r*).

## 4. Results and discussion

### 4.1. Effect of the Néel surface anisotropy

**Figure 4:**
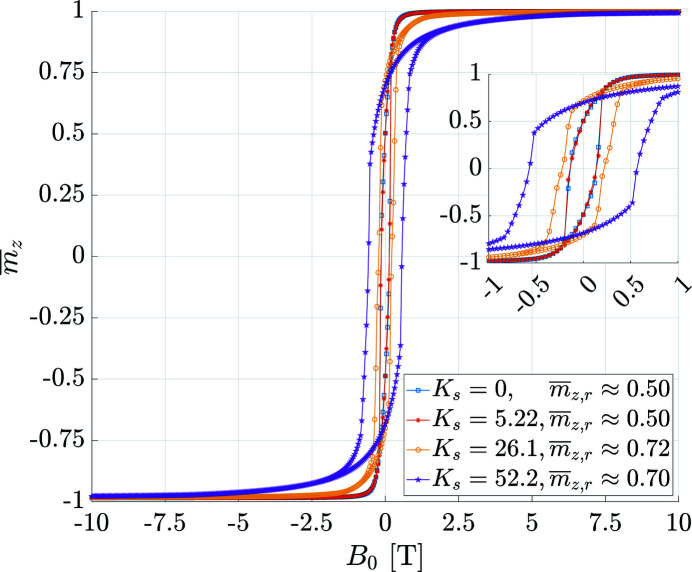
The computed normalized magnetization 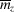 [compare equation (9)] of an ensemble of randomly oriented spherical nanomagnets for different values of the surface anisotropy constant *K*_s_ (in units of 10^−23^ J atom^−1^, see inset). The particle diameter is *D* = 10 nm and the remanence values are indicated in the inset.

Fig. 3 ▸ displays as an example the spin structures of a 5 nm-sized spherical nanomagnet for the cases of a small and large surface anisotropy constant *K*_s_, and Fig. 4 ▸ shows computed hysteresis curves for an ensemble of randomly oriented 10 nm-sized nanomagnets. As expected, increasing *K*_s_ results, for a given particle size, in a progressive surface spin disorder which propagates into the bulk of the nanomagnet. The effect of an enhanced *K*_s_ also becomes visible in the magnetization curves via an increased coercivity *H*_c_ and remanence *m*_r_. For *K*_s_ = 0 and dominant exchange, we recover the well known results from the Stoner–Wohlfarth model (Usov & Peschany, 1997 ▸), *i.e.* we find a reduced remanence of *m*_r_ = 0.5 and a coercivity of 

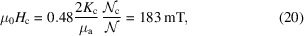

where 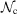 denotes the number of atoms belonging to the particle’s core. Note that for the case of a strong surface anisotropy [Fig. 3 ▸(*b*)], the mean magnetization at remanence deviates strongly from the core anisotropy axis (parallel to **e**_*z*_), which is in contrast to the case of weak anisotropy [Fig. 3 ▸(*a*)]. This observation is in agreement with the analytical calculations by Garanin & Kachkachi (2003 ▸) who predicted the emergence of an effective anisotropy of cubic symmetry for dominant *K*_s_. Therefore, with increasing *K*_s_ we initially observe in Fig. 4 ▸ an increase in the remanence. However, for the largest *K*_s_, the reduced remanence again decreases slightly from 0.72 to 0.70. We believe that this observation is due to the disordering effect of the surface anisotropy beyond a certain critical *K*_s_.

**Figure 5:**
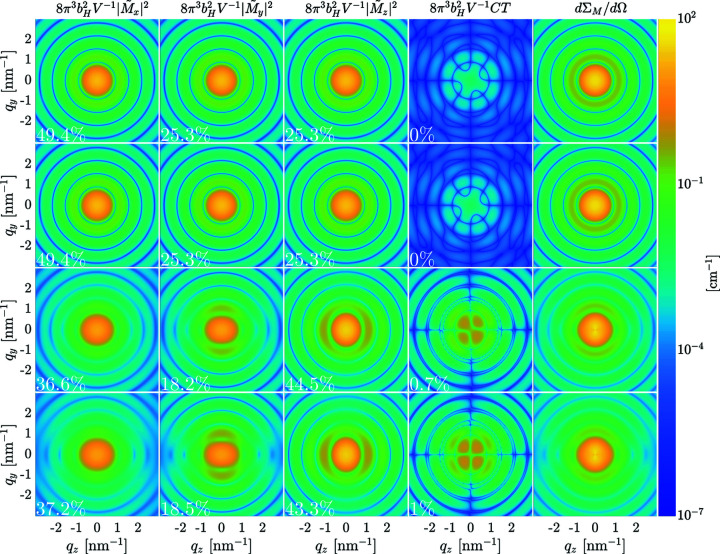
Decryption of the two-dimensional magnetic SANS cross section dΣ_M_/dΩ in the remanent state (*B*_0_ = 0 T) into the individual magnetization Fourier components 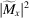 , 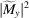 and 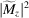 , and CT = 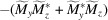 (see insets) (logarithmic colour scale). Note that the respective Fourier components are multiplied by the constant 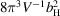 (in order to have the same units as dΣ_M_/dΩ), but not by the trigonometric functions in the expression for dΣ_M_/dΩ [see equation (13)]. The % values specify the fraction of the respective Fourier component of the total dΣ_M_/dΩ [see equation (21) and associated discussion in the main text]. The CT (and hence the corresponding η_α_) can take on negative values, but in this figure we show (due to the chosen logarithmic colour scale) the absolute value of the CT. The data correspond to an ensemble of randomly oriented 10 nm-sized nanomagnets. The *K*_s_ values for each row are (first row) *K*_s_ = 0, (second row) *K*_s_ = 5.22 × 10^−23^ J atom^−1^, (third row) *K*_s_ = 26.1 × 10^−23^ J atom^−1^ and (fourth row) *K*_s_ = 52.2 × 10^−23^ J atom^−1^.

**Figure 6:**
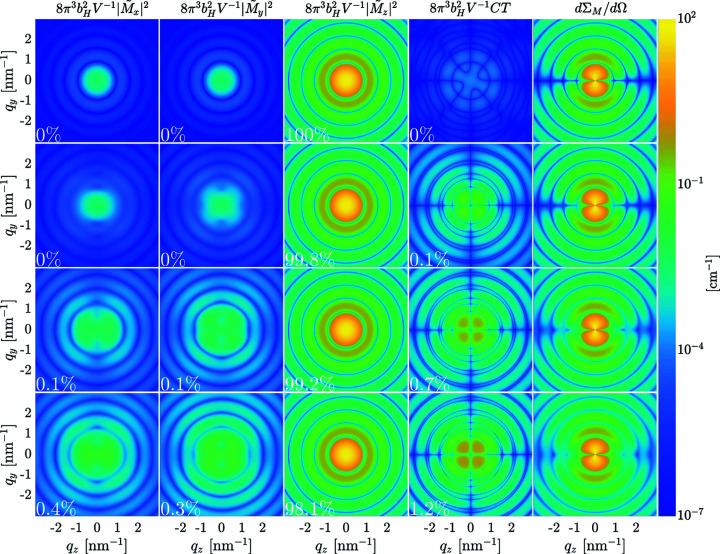
The same as Fig. 5 ▸, but for *B*_0_ = 10 T.

Fig. 5 ▸ displays the two-dimensional magnetic SANS cross section dΣ_M_/dΩ of an ensemble of 10 nm-sized nanomagnets in the remanent magnetization state, along with the individual Fourier components 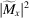 , 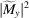 and 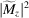 , and the cross term CT = 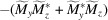 [see equation (13)]. Fig. 6 ▸ shows the corresponding plots at a (nearly) saturating field of *B*_0_ = 10 T. We emphasize that the depicted scalar functions represent projections of the corresponding three-dimensional quantities onto the *q*_*y*_ *q*_*z*_ detector plane at *q*_*x*_ = 0 (see Fig. 2 ▸). The surface anisotropy constant *K*_s_ increases from the top to the bottom row in Figs. 5 ▸ and 6 ▸. It is seen that, generally, all the Fourier components contribute to dΣ_M_/dΩ.

Near saturation (Fig. 6 ▸), dΣ_M_/dΩ is dominated for all values of *K*_s_ by the isotropic (θ-independent) 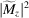 Fourier component and exhibits the characteristic 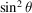 anisotropy with two maxima along the vertical direction [compare equation (13)]. Increasing *K*_s_ enhances the contributions of both transverse Fourier components 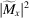 and 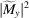 and of the CT. Moreover, the latter contributions develop a pronounced angular anisotropy with increasing *K*_s_.

At remanence (Fig. 5 ▸), dΣ_M_/dΩ and all the Fourier components are isotropic for small values of *K*_s_ and become progressively more anisotropic with increasing *K*_s_. For instance, 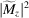 is initially isotropic and develops a pronounced angular anisotropy that is elongated along the *q*_*y*_ direction for larger *K*_s_. The CT also develops an anisotropy with increasing *K*_s_, with maxima roughly along the detector diagonals. An anisotropic magnetic SANS cross section at zero applied magnetic field of an ensemble of randomly oriented nanoparticles has also been found in the micromagnetic continuum simulations of Vivas *et al.* (2020 ▸). These authors did not consider the Néel surface anisotropy but included the magnetodipolar interaction.

To quantify the fraction of the individual Fourier components in equation (13) relative to the total magnetic SANS cross section dΣ_M_/dΩ, we compute the following dimensionless quantity: 

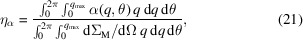

where α(*q*, θ) is, respectively, given by 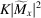 , 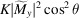 , 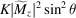 and 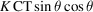 , with 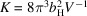 . *q*_max_ is taken as 10 nm^−1^. The corresponding numbers are given as % values in Figs. 5 ▸ and 6 ▸, and we note that the contribution related to 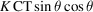 can be positive as well as negative, in contrast to the other three contributions which are strictly positive. Using the inequality 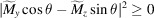 , it can easily be shown that the contribution 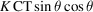 is, however, always smaller than the sum of the other terms (as it must be). We emphasize that the colour-coded plots in Figs. 5 ▸ and 6 ▸ show the respective Fourier components without the trigonometric functions in equation (13), whereas the quantities η_α_ do contain the trigonometric terms. For *K*_s_ = 0 and zero field, the contributions of 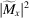 , 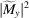 and 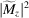 to dΣ_M_/dΩ are approximately equal (while CT = 0). This can be understood by noting the isotropy of these functions and by taking into account the trigonometric terms 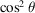 (for 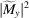 ) and 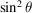 (for 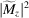 ), which yield a factor of 1/2 on azimuthal averaging [θ integration, compare equation (21)]. At remanence, the results for the smallest nonzero *K*_s_ are nearly identical to the data for *K*_s_ = 0 (compare Fig. 4 ▸).

**Figure 7:**
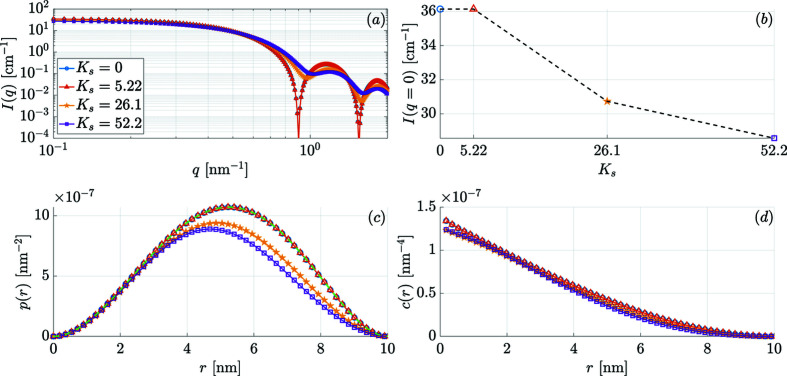
The effect of the surface anisotropy constant *K*_s_ (in units of 10^−23^ J atom^−1^, see inset) on (*a*) the azimuthally averaged magnetic SANS cross section *I*(*q*) = (dΣ_M_/dΩ)(*q*) (log–log scale), (*b*) the value of the magnetic SANS cross section at the origin, *I*(*q* = 0) versus *K*_s_, (*c*) the pair-distance distribution function *p*(*r*) and (*d*) the correlation function *c*(*r*). The data correspond to the remanent state (*B*_0_ = 0 T) and the nanomagnets’ diameter is 10 nm. The green dashed line in panel (*c*) displays the analytical pair-distance distribution function for the case of a uniformly magnetized spherical particle [proportional to equation (18)], where the magnitude is normalized to the maximum value from the numerical simulation in the case *K*_s_ = 0.

**Figure 8:**
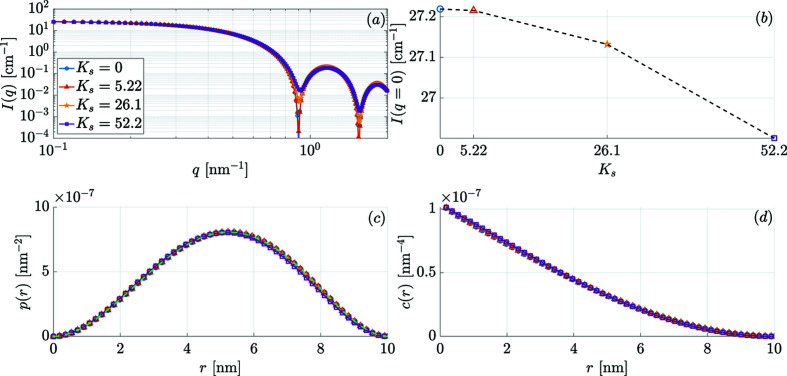
The same as Fig. 7 ▸, but for *B*_0_ = 10 T.

The effect of increasing *K*_s_ on the 2π azimuthally averaged *I*(*q*) = dΣ_M_/dΩ and on *p*(*r*) and *c*(*r*) is shown in Fig. 7 ▸ for the remanent state and in Fig. 8 ▸ for *B*_0_ = 10 T. With increasing spin disorder (induced by an increasing *K*_s_) we observe in Fig. 7 ▸(*a*) that (i) the characteristic form-factor oscillations of *I*(*q*) are progressively damped and (ii) the extrema in *I*(*q*) shift to larger *q* values due to the reduced coherent magnetic size of the particle. Generally, in experimental situations, the smearing of form-factor oscillations is related to the effect of a particle-size distribution function and/or experimental resolution. Therefore, when data such as those in Fig. 7 ▸(*a*) are fitted to a set of single-domain particles with a distribution of sizes, rather than to a set of non-uniformly magnetized particles that all have the same size, an erroneous value for the particle size may result. At (quasi)saturation [Fig. 8 ▸(*c*)] and for small *K*_s_ at remanence [Fig. 7 ▸(*c*)], we recover the analytically known expressions for *I*(*q*), *p*(*r*) and *c*(*r*) for uniformly magnetized spherical particles [equations (15) and (18)]. We have also plotted in Figs. 7 ▸(*b*) and 8 ▸(*b*) the *K*_s_ dependence of the *q* = 0 extrapolated value of *I*(*q*). The quantity *I*(*q* = 0) is directly proportional to the static susceptibility χ(*q* = 0) (as it can be measured with a magnetometer), which itself is proportional to the mean-square fluctuation of the magnetization per atom (Marshall & Lowde, 1968 ▸). We see that, as expected, the increase in *K*_s_ has a large effect on χ(0), whereas the reduction is relatively small at 10 T. We also refer to Fig. 10 in Appendix *A* [App appa], where the results for the SANS observables are shown for the other possible sign combinations of the core and surface anisotropy constants.

### 4.2. Effect of a particle-size distribution

In SANS experiments on nanoparticles one always has to deal with a distribution of particle sizes and shapes. The size of a particle has an important effect on its spin structure, *e.g.* smaller particles generally tend to be nearly uniformly magnetized (due to the dominant role of the exchange interaction), whereas larger particles may exhibit highly inhomogeneous spin structures (due to the magnetodipolar interaction) (Vivas *et al.*, 2020 ▸). It is therefore also of interest to study the influence of a distribution of particle sizes on the magnetic SANS observables [dΣ_M_/dΩ, *p*(*r*), *c*(*r*)]. This has been done using a log-normal probability distribution function, which is defined as (Krill & Birringer, 1998 ▸)

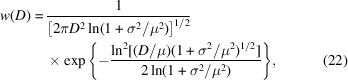

where μ denotes the expectation value and σ^2^ is the variance, such that

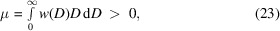




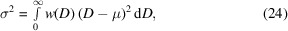

where the corresponding median 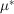 is determined by the relation

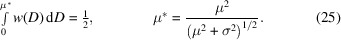

For given values of μ and σ, the average magnetic SANS cross section 〈…〉 is computed as

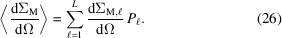


*P*_ℓ_ denotes the probability related to the particle-size class *D*_ℓ_ = 2*R*_ℓ_ (diameter), which is computed as

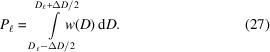

**Figure 9:**
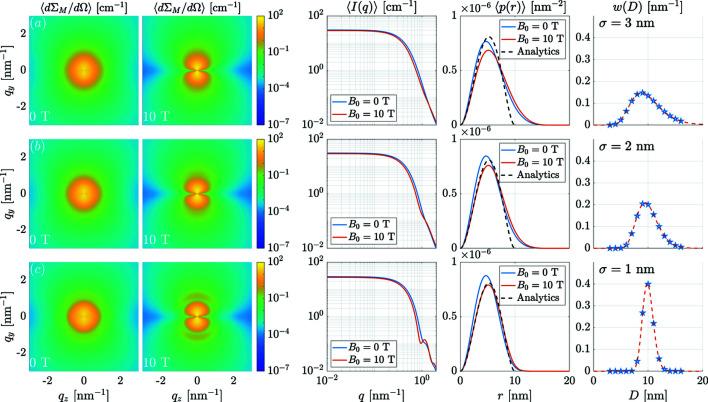
The effect of a log–normal particle-size distribution function on the SANS observables (*K*_s_ = 52.2 × 10^−23^ J atom^−1^). Shown are the two-dimensional 〈dΣ_M_/dΩ〉, the corresponding azimuthally averaged 〈*I*(*q*)〉, the pair-distance distribution functions 〈*p*(*r*)〉 and the particle-size distributions *w*(*D*) for σ values of (*a*) 3 nm, (*b*) 2 nm and (*c*) 1 nm. These σ values correspond to [ln(1 + σ^2^/μ^2^)]^1/2^ values of, respectively, 0.29, 0.20 and 0.10. The nanoparticles’ mean diameter (expectation value) was chosen as μ = 10 nm in each case. The data correspond to the remanent (*B*_0_ = 0 T) and saturated (*B*_0_ = 10 T) magnetization states. The discrete particle-size classes are defined by the particle diameters *D* = 3–16 nm with an equidistant step size of Δ*D* = 1 nm. The black dashed 〈*p*(*r*)〉 curves are the analytically known solutions for uniformly magnetized spheres of size μ = 10 nm in the fully saturated state.

**Figure 10:**
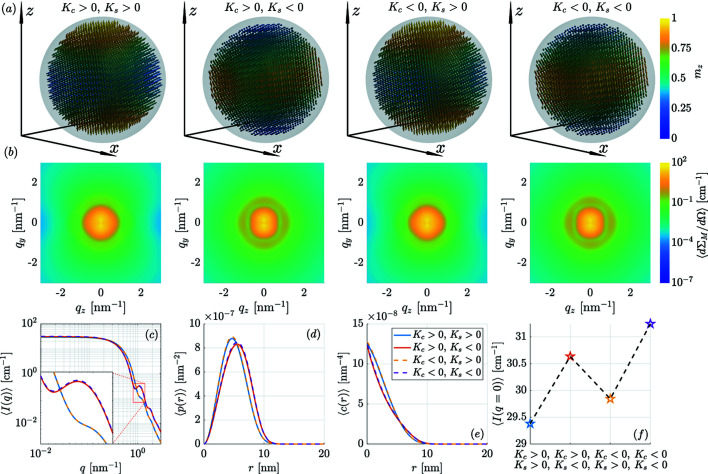
Selected spin structures and results for the SANS observables for different signs of the anisotropy constants (see legends, |*K*_c_| = 3 × 10^−24^ J atom^−1^ and |*K*_s_| = 52.2 × 10^−23^ J atom^−1^). The case *K*_c_ > 0 and *K*_s_ > 0 from Fig. 9 ▸(*c*) is included for completeness. (*a*) Snapshots of three-dimensional real-space magnetization configurations (particle size 10 nm). (*b*) The corresponding two-dimensional 〈dΣ_M_/dΩ〉. (*c*) The azimuthally averaged 〈*I*(*q*)〉. (*d*) The pair-distance distribution functions 〈*p*(*r*)〉. (*e*) The correlation functions 〈*c*(*r*)〉. (*f*) The value of the magnetic SANS cross section at the origin, 〈*I*(*q* = 0)〉. The data in panels (*b*)–(*f*) correspond to an ensemble of randomly oriented nanoparticles with a mean diameter of μ = 10 nm and σ = 1 nm in a remanent magnetization state (*B*_0_ = 0 T) after prior saturation. The inset in panel (*e*) specifies the signs of the anisotropy constants for panels (*c*)–(*f*).

Fig. 9 ▸ summarizes the results obtained for the magnetic SANS cross section and correlation function. As expected, one observes a smearing of the SANS signal with increasing standard deviation σ of the distribution, which becomes particularly visible in the azimuthally averaged 〈*I*(*q*)〉 curves via the suppression of the form-factor oscillations. The angular anisotropy of the SANS cross section in the remanent state, which can be seen as a characteristic signature of the Néel surface anisotropy (compare also the lowest row in Fig. 5 ▸), becomes less pronounced for large σ. An increasing applied field suppresses the internal spin disorder and in this way increases the coherent magnetic sizes of the nanoparticles, so that the maximum of 〈*p*(*r*)〉 shifts to larger distances. At the same time, an increasing field also suppresses fluctuations in the local magnetizations relative to the mean directions, which then results in a reduction in the magnitude of 〈*p*(*r*)〉.

Up to this point, we have exclusively considered the case where both anisotropy constants are positive, *i.e.* *K*_c_ > 0 and *K*_s_ > 0 (for fixed magnitudes). In Appendix *A* [App appa] we also show the results for the SANS observables for the other possible sign combinations of the anisotropy constants. There, we can see that the angular anisotropy and the *q* dependence of 〈dΣ_M_/dΩ〉 may be taken as an indication for distinguishing between the cases of positive and negative *K*_s_.

In contrast to our accompanying analytical study (Adams *et al.*, 2022 ▸), which is based on the linearization of Brown’s static equations of micromagnetics, the present numerical work takes into account the full nonlinearity of the underlying equations for the spin dynamics via the Landau–Lifshitz equation. Therefore, the analytical approach is only valid for weak surface anisotropy, while the numerical approach considers surface anisotropy of arbitrary strengths. In both studies, the dipolar interaction is neglected, which is related to its mathematical complexity and the enormous numerical effort to include it in atomistic simulations of large particles.

## 5. Conclusions and outlook

We have studied the spin structure and magnetic neutron scattering signal of an ensemble of randomly oriented spherical nanomagnets using the Landau–Lifshitz equation, with particular focus on the Néel surface anisotropy. Taking into account the isotropic exchange interaction, an external magnetic field, a uniaxial magnetic core anisotropy and the Néel surface anisotropy, we compute the magnetic small-angle neutron scattering cross section and the pair-distance distribution function from the obtained equilibrium spin structures. The numerical results are compared with the well known analytical expressions for uniformly magnetized particles. With increasing internal spin disorder (increasing surface anisotropy *K*_s_), the pair-distance distribution function (at remanence) exhibits a systematic shift of its maximum to smaller *r* values and the total magnetic SANS cross section develops a characteristic anisotropic scattering pattern. The strength of the simulation methodology is that the field evolution of the individual Fourier components and their contribution to the magnetic SANS signal can be monitored. Atomistic and micromagnetic continuum simulations have contributed and will continue to contribute to the fundamental understanding of magnetic SANS.

In our future work, we will focus on the inclusion of both the intraparticle and interparticle dipole–dipole energy and the Dzyaloshinskii–Moriya interaction, which will give rise to more complicated spin textures (*e.g.* vortex-type structures), in particular for larger particle sizes. Moreover, it is of interest to compare the Néel anisotropy with other phenomenological expressions for the surface anisotropy, such as energy densities of the type 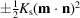 , where **n** is the normal unit vector to the surface (instead of **u**_*ij*_), or with the case of a truly random surface anisotropy, where **u**_*ij*_ are random vectors. In this regard, the present first atomistic simulations may be considered as the starting point towards a more complete description of magnetic SANS.

The supporting information to this paper features a video that displays the SANS observables during the magnetization-reversal process for the case of a strong surface anisotropy.

## Supplementary Material

SANS observables during magnetization reversal for nanoparticles with strong surface anisotropy. DOI: 10.1107/S1600576722008949/in5071sup1.mp4

## Appendix A Summary of results for different signs of the anisotropy constants

In the main text, we have exclusively considered the case that both anisotropy constants are positive, *i.e.* *K*_c_ > 0 and *K*_s_ > 0. Here, for completeness, we also display in Fig. 10 ▸ the results for the SANS observables for the cases of *K*_c_ > 0 and *K*_s_ < 0, *K*_c_ < 0 and *K*_s_ > 0, and *K*_c_ < 0 and *K*_s_ < 0. By comparison with equation (3) it is seen that changing the sign of the core anisotropy constant *K*_c_ from positive to negative changes the orientation from easy axis to easy plane. Likewise, changing the sign of *K*_s_ from positive to negative favours the **m**_*i*_ ∥ **u**_*ij*_ orientation (which we term the tangential orientation) over the **m**_*i*_ ⊥ **u**_*ij*_ (radial) orientation. The magnitudes of the anisotropy constants are |*K*_c_| = 3 × 10^−24^ J atom^−1^ and |*K*_s_| = 52.2 × 10^−23^ J atom^−1^. A log-normal particle-size distribution function with a mean diameter of μ = 10 nm and a width of σ = 1 nm has been assumed.

It is seen in Fig. 10 ▸ that the sign change of *K*_s_ has the largest effect on the SANS observables. The surface spin structure [Fig. 10 ▸(*a*)] changes from a more radial spin orientation for *K*_s_ > 0 to a more tangential orientation for *K*_s_ < 0. The corresponding angular anisotropy of the two-dimensional 〈dΣ_M_/dΩ〉 [Fig. 10 ▸(*b*)] changes from a horizontally elongated (*K*_s_ > 0) to a vertically elongated pattern (*K*_s_ < 0). The sign change of *K*_s_ also manifests in the 〈*p*(*r*)〉 and 〈*c*(*r*)〉 functions, but the most prominent effect is seen in 〈*I*(*q*)〉 [Fig. 10 ▸(*c*)], where at *q* ≅ 0.8–1.4 nm^−1^ the functional dependency of 〈*I*(*q*)〉 is distinctly different, more shoulder-like for *K*_s_ > 0 to more peak-like for *K*_s_ < 0 [see inset in Fig. 10 ▸(*c*)]. This feature could be taken as an indication for distinguishing between the two cases of *K*_s_ > 0 and *K*_s_ < 0.
